# Bullosis diabeticorum: a treatment conundrum

**DOI:** 10.1186/1757-1146-4-S1-P12

**Published:** 2011-05-20

**Authors:** Peta Craike

**Affiliations:** 1 Podiatry Lecturer , School of Health Sciences, University of Newcastle, Ourimbah, NSW, 2258, Australia

## 

Bullosis diabeticorum is an infrequent but significant complication of diabetes Mellitus most commonly affecting the hands and feet. These rapidly developing bullous lesions mostly occur in patients with long standing diabetes and neuropathy. The pathophysiology of this condition remains unknown. Despite reasonably low rates of occurrence this complication potentially has significant and serious ramifications for foot health and creates a treatment conundrum. This case study demonstrates the serious nature of seemingly innocuous presentations in management of the diabetic foot. A 76-year-old man presents to the high-risk foot clinic for treatment of a suspected Charcot foot. He has a complex medical history, which includes Type 2 diabetes, hypertension, congestive cardiac failure, hypercholesterolemia, and Gastro-esophageal reflux disease. The patient undergoes various testing to aid in diagnosing a Charcot foot, such as skin temperature testing, X-ray and bone scans. Fortunately he was not diagnosed with a Charcot foot. During a routine follow-up consult he presents with clear, serous filled blisters which have spontaneously appeared. They are in non weight-bearing areas, and the patient does not recall any trauma to the area. The blisters appear consistent with bullosis diabeticorum. There are no set criteria for appropriate treatment of blistering in these cases. Treatment options were to either leave blisters intact or de-roof them, and their treatment raises many questions. Intact blisters were left intact to maintain a sterile field; broken blisters were de-roofed to prevent infection as per normal protocol for any form of blister management. The healing outcomes were compared, with no significant difference noted. However, after healing was achieved, the patient returned to the clinic, weeks later, with another episode of blistering. The patient recalled a similar history to the first episode, with no traumatic injury to the site, and the blisters occurring overnight. The same treatment protocol was followed. However, on this occasion, the blisters did not heal as successfully, and the patient developed osteomyelitis, and subsequently suffered multiple digital amputations as a result. This case demonstrates that successful wound care can be difficult on a patient with diabetes and associated complications, such as neuropathy, peripheral vascular disease, and an increased susceptibility to infection.

